# Standardized chemical synthesis of *Pseudomonas aeruginosa* pyocyanin

**DOI:** 10.1016/j.mex.2014.07.001

**Published:** 2014-07-08

**Authors:** Rajkumar Cheluvappa

**Affiliations:** Department of Medicine, St. George Clinical School, University of New South Wales, Sydney, NSW, Australia

**Keywords:** Pyocyanin, *Pseudomonas aeruginosa*, Liver sinusoidal endothelial cells, Phenazine methosulfate, Glutathione

## Abstract

Preparation of the toxin pyocyanin from the bacterium *Pseudomonas aeruginosa* is an exacting procedure. Pyocyanin is expensive to commercially purchase. The sellers do not give out the extraction procedure. Classically, pyocyanin preparation involves complicated multi-step *P. aeruginosa* culturing and solvent transfer extractions. The chemical synthesis first used (1979) has not been adequately described. We devised an easily reproducible protocol which consistently decreases the time taken for synthesis, extraction and purification of pyocyanin, and increases the pure pyocyanin proportion produced.

Our procedure:

• Involves more purification steps (chloroform/methanol/acidification/alkalinization).
• Starts with a different pH (7.4 instead of 7), and lesser concentration of phenazine methosulfate; and retrenches a rotary evaporation step.
• Removes 2 lyophilization steps, and entails different solvent proportions for thin layer chromatography.

As we have extracted pyocyanin both from *P. aeruginosa* cultures, and via chemical synthesis; we know the procedural and product-quality differences. We endorse the relative ease, safety, and convenience of using the chemical synthesis described here. Crucially, our “naturally endotoxin-free” pyocyanin can be extracted easily without using infectious bacteria.

## Graphical abstract

**Figure:**
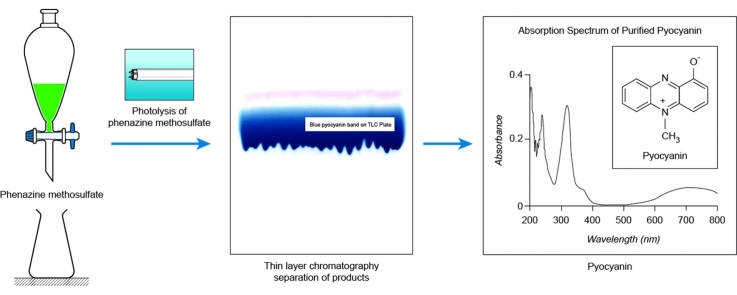

## Materials

### Reagents

1. Phenazine methosulfate or PMS (Laboratory reagent grade) – P9625 (Sigma-Aldrich Pty. Ltd, Sydney, Australia)
2. Nitrogen (Industrial grade) – 032 (BOC gases, Sydney, Australia)
3. Chloroform (Laboratory reagent grade) – 132950 (Sigma-Aldrich Pty. Ltd, Sydney, Australia)
4. Hexane (Laboratory reagent grade) – 296090 (Sigma-Aldrich Pty. Ltd, Sydney, Australia)
5. Methanol (Laboratory reagent grade) – 322415 (Sigma-Aldrich Pty. Ltd, Sydney, Australia)
6. Millipore water
7. Stock HCl (Laboratory reagent grade) – 320331 (Sigma-Aldrich Pty. Ltd, Sydney, Australia)
8. TRIS–HCl (Laboratory reagent grade) – T3253 (Sigma-Aldrich Pty. Ltd, Sydney, Australia)
9. NaOH pellets (Laboratory reagent grade) – S8045 (Sigma-Aldrich Pty. Ltd, Sydney, Australia)

### Equipment

1. Nitrogen tank, pressure gauge with outlet, gas tubes
2. pH meter/probe
3. Fluorescent tube light – Phillips TLD 18 W/54 (TIS.958-2533 and TIS.236-2533)
4. Separation funnels
5. Pear-shaped glass flasks
6. Fume hood
7. Pasteur pipettes
8. Tape and fasteners
9. Centrifuge (5000 rpm – capable with 50 ml polypropylene tube-holders)
10. 50 ml polypropylene tubes
11. Polypropylene syringe
12. Type EH 0.5 m filters
13. Thin Layer Chromatography (TLC) Plates – TLC plates from Merck (HPTLC Pre-coated Silica Gel 60 Plates)
14. Glass gas cage without paper lining
15. A4 size paper
16. Microwave oven
17. Desiccator
18. Sample applicator (Camag Nanomat), applicator syringe, 1 ml and 5 ml applicator glass tips
19. Computer scanner
20. Sterile scalpel blades
21. Small glass tubes, and refrigerated centrifuge with small slots to hold the small glass tubes
22. Shimadzu Spectrophotometer with deuterium lamp emitting UV light (wavelength ranging from 200 to 350 nm) and a halogen lamp (wavelength ranging from 350 to 800 nm – visible light)
23. Quartz cuvettes

## Method details

Our “customization” of the original method [1] is succinctly posited in Table 1. Most of the steps are different, and hence the preference of the Table 1 summary over stepwise annotation in this section.

**Fig. 1:**
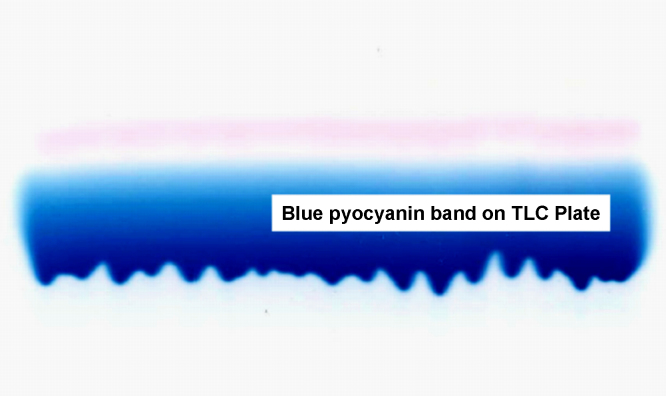
TLC purification of pyocyanin.

**Fig. 2:**
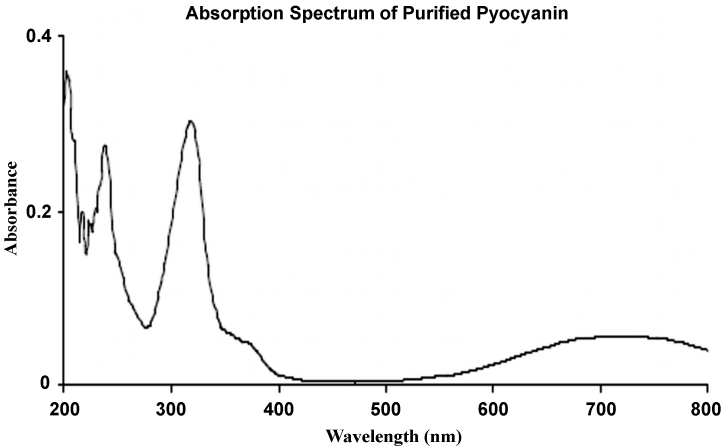
Spectrophotometric estimation of pyocyanin concentration.

1. 100 mg PMS added to 100 ml of 10 mM TRIS–HCl in a 100 ml capacity thin-stemmed round bottomed glass flask
2. pH to 7.4 using 0.5 M NaOH
3. The reaction mixture kept 25 cm from daylight Phillips TLD 18 W/54 fluorescent tube light for 2.5 h.
4. Chloroform added to the reaction mixture in a separation funnel kept in a fume hood
5. The lower chloroform (organic) phase with pyocyanin transferred to a pear-shaped flask
6. Nitrogen bubbled through the contents of the pear-shaped flask till a blue pyocyanin sludge remains after all the chloroform has been evaporated
7. Blue pyocyanin sludge resuspended in 50 ml chloroform
8. Pyocyanin chloroform solution acidified with 50 ml of 0.1 M HCl – pyocyanin becomes red
9. 50 ml of chloroform added
10. A few drops of 500 mM NaOH added to convert red pyocyanin to blue pyocyanin
11. Chloroform extraction done twice with 50 ml chloroform
12. Pyocyanin chloroform solution kept at −20 °C freezer overnight
13. Chloroform evaporated by gentle nitrogen insufflation
14. Pyocyanin resuspended in small amounts of more chloroform
15. Pyocyanin chloroform (concentrated) solution transferred to a vertical Pyrex glass tube
16. Chloroform evaporated
17. Hexane wash done by adding hexane (pyocyanin is insoluble in hexane), swirling the tube, and aspirating the hexane out
18. Chloroform (1 ml) added to pyocyanin
19. Hexane (5–8 ml) added SLOWLY, DROP by DROP to the pyocyanin chloroform solution
20. 10 min waiting period mandated
21. Pyocyanin crystallized automatically
22. Pyocyanin in hexane and chloroform in 50 ml polypropylene tubes is centrifuged at 5000 rpm for 10 min
23. Supernatant pipetted out from the 50 ml tubes and discarded
24. Lower phase with the pyocyanin crystals transferred to a polypropylene syringe (without a piston) with its tip compactly fitted into a the top nozzle of a filter apparatus containing a type EH 0.5 m filter
25. Pyocyanin crystals trapped by filter
26. Methanol elution into a screw-capped glass bottle done
27. Methanol evaporated by nitrogen bubbling
28. Small amount of methanol used to dissolve the pyocyanin
29. Pyocyanin methanol solution kept at −20 °C freezer overnight
30. Methanol evaporated by nitrogen bubbling
31. Pyocyanin reconstituted in 0.5 ml of chloroform
32. Silica glass TLC plate activated:
  (i) Excess silica from 3 edges scraped out
  (ii) TLC plate kept vertically in 10 ml methanol in a glass cage without paper lining. The most jagged/damaged edge placed inferior and in contact with methanol in the glass cage.
  (iii) Glass plate used to cover the glass cage
  (iv) TLC plate is taken out of the methanol (and the glass cage) as soon as the solvent (methanol) front reaches 2 cm from the top edge (of the TLC plate)
  (v) TLC plate kept outside the glass cage, inside the hood, for 10 min
  (vi) TLC plate kept on a folded A4 size paper and heated with silica side up in a microwave at minimum power setting for 5 min
  (vii) TLC plate heated in the microwave at power setting 3 for 10 more min
  (viii) TLC plate kept in the dark or inside a desiccator till use
33. Sample applicator (Camag Nanomat) fitted with a applicator syringe fitted with a 1 μl (preferably) or a 5 μl glass tip, utilized to apply 0.25 ml pyocyanin chloroform solution to the silica part of the silica glass TLC plate
34. TLC plate with loaded pyocyanin kept in chloroform methanol mixture (12.5 ml: 12.5 ml) inside a glass cage with a paper lining
35. TLC plate removed from the glass cage when the solvent front reaches 2 cm from the top edge
36. TLC plate computer-scanned and image saved (Fig. 1)
37. Silica layer with the pyocyanin carefully scraped from the glass part of the TLC plate
38. Scraped silica with pyocyanin dissolved in 2 ml methanol in a screw capped glass bottle
39. Silica pyocyanin methanol solution transferred to small glass tubes (compatible in the slots of the centrifuge to be described soon)
40. Glass tubes centrifuged twice in the centrifuge available inside the walk-in refrigerator (the sealing lid is not shut, only the topmost trap lid is shut)
41. Quantification using spectrophotometer:
  (i) The calibrations should be as follows:
    • Measuring Mode: Abs
    • Recording Range: Low 0.0 to High 0.5
    • Wavelength Range (nm): 800 to 200 nm
    • Scan Speed: Fast
    • Sampling Interval nm: 1
42. Spectrophotometric Estimation of Pyocyanin Concentration (Fig. 2)
  (i) Use quartz cuvettes of 1 cm light path (1 ml capacity)
  (ii) Use 2 cuvettes filled with methanol in each to blank (baseline). Place cuvette 1 in the proximal slot and cuvette 2 in the distal slot of the spectrophotometer
  (iii) Blanking (baselining) is done by clicking Baseline
  (iv) To check whether the blanking was done properly, click Start. The absorbance value should be 0
  (v) Dilute pyocyanin in methanol to 1:100 dilution (10 ml:990 ml) in cuvette 1 (from proximal slot in the spectrophotometer). This is the sample cuvette. Place the sample cuvette in the proximal slot in the spectrophotometer
  (vi) Click Start
  (vii) Pyocyanin typically peaks at 718 nm, 318 nm and 239 nm (Fig. 2). The absorption values at these spectra are noted
43. Calculation of Molar Concentration using Spectrophotometer Absorption Data
  (i) Adjust the concentration of pyocyanin (by drying the methanol using nitrogen surface insufflation in a Fume Hood and/or by adding more methanol) till a 1 mM concentration is obtained. The millimolar concentration of pyocyanin solution in methanol can be determined as follows
  (ii) Please note that extinction coefficients depend on the solvent used and the specific wavelength of absorption spectra
    • Different solvents have different Extinction Coefficients
    • Different Absorption wavelengths have different Extinction Coefficients
  (iii) Absorbance = Molar Concentration × Light path × Extinction Coefficient
  (iv) *A* = *E* × *L* × *C* = *E* × 1 *Cm* × *C* = *E* × *C*
  (v) Therefore the Molar Concentration *C* = *A*/*E* × Dilution Factor = *A*/*E* × 100
  (vi) Obtain the absorption values for the following wavelengths:
    • 718 nm
    • 318 nm
    • 239 nm
  (vii) The typically used absorption wavelength of pyocyanin for calculation of Molar concentration is at 318 nm, because that is where the highest peak is seen
  (viii) The Extinction Coefficient *E* of pyocyanin in methanol at 318 nm is 30,199.5
  (ix) Therefore the millimolar concentration of pyocyanin is 1 mM

## Additional information

The Gram-negative bacterium *P. aeruginosa* is the most common cause of chronic and recurrent lung infections in patients with cystic fibrosis whose sputa contain copious quantities of *P. aeruginosa* toxin, pyocyanin. Pyocyanin is a blue-green, phenazine pigment, which triggers tissue damage mainly via its redox cycling, and induction of reactive oxygen species [2]. Pyocyanin is expensive to purchase commercially, and the sellers are reticent in giving out the minutiae of the extraction procedure. The classical method of preparation involves multi-step bacterial culturing and solvent transfer extraction, as first spelt out [3]. A modified chemical synthesis was first presented (1979) with a few details in the journal “Analytical Biochemistry” [1]. However, the methodology was not described in detail, and the technical intricacies not posited. This paper does exactly that. We present an extensively revamped version of the modified procedure, spelling out the steps in detail.

The blue band on the TLC plate is formed by pyocyanin (Fig. 1), and the pink band by impurities. The final purified pyocyanin peaked at 718 nm, 318 nm and 239 nm (Fig. 2), typical for pyocyanin synthesized from bacterial cultures.

We have extracted pyocyanin by both microbial extraction and chemical synthesis, and we acknowledge and endorse the relative ease, safety, and convenience of using the chemical synthesis described here. Our changes to the previous procedure involve the following parameters (Table 1):

(i) Initial concentration of PMS used
(ii) Starting pH
(iii) Photolysis time
(iv) Rotary evaporation
(v) Lyophilization of photolysed products
(vi) Sequential acidification/alkalinization, and chloroform extraction-purification
(vii) Hexane precipitation from chloroform, filtration, methanol elution from filter TLC plate
(viii) Chloroform:methanol proportion for TLC plate equilibration
(ix) Solvent for pyocyanin loading on TLC plate
(x) Chloroform:methanol proportion for pyocyanin elution
(xi) Lyophilization after pyocyanin elution
(xii) Pyocyanin yield from TLC plate Purification steps
(xiii) Total time for extraction and purification

With the pyocyanin extracted using the protocol above, we had generated the following data (previously published).

(1) Pyocyanin reacted with reduced glutathione (GSH) non-enzymatically at 37 °C resulting in the production of red-brown products (480 nm absorption peaks) [2]. The reaction between pyocyanin and GSH was concentration-dependent on reduced glutathione, but not on pyocyanin. Catalase circumvented the reaction. The presence of a previously unidentified non-enzymatic GSH-dependent metabolic pathway for pyocyanin has thus been identified. The formation of H_2_O_2_ as an intermediate and the thiol group in GSH is crucial to this reaction.
(2) Pyocyanin treatment induced a dose-dependent reduction in fenestrations in isolated LSECs [4]. In isolated LSECs, pyocyanin induced a catalase-preventable loss of sieve plate organization with significant reductions in LSEC porosity. In the intact liver [4], within 30 min of intraportal injection of a pathophysiologically relevant dose of pyocyanin, there was a significant reduction in porosity

The “original” method, and the most common method of pyocyanin preparation involves complicated multi-step solvent extractions from *P. aeruginosa* cultures [3]. We present a clear-cut chemical synthesis procedure, positing the smallest of steps in exquisite detail. Our exhaustively overhauled procedure (Table 1) markedly decreased the total time taken for the synthesis, extraction and purification [1]. Importantly, our procedure substantially increased the proportion of pure pyocyanin obtained during the final step, although it entailed more purification steps. It also cut short a rotary evaporation step and multiple lyophilization steps at 2 different points of time. Crucially, pyocyanin could be synthesized with ease from phenazine methosulfate, without using infectious bacteria. It is also “naturally endotoxin-free”. We hope that this paper will benefit interested scientists immensely.

## Source of support

National Health and Medical Research Council (NHMRC).

## Conflicts of interest

None to report.

## Figures and Tables

**Table 1:** Procedural differences between the original procedure and our procedure.

	Procedure step (S)	First description [1]	Our protocol [2,4–7]
1	Number of variations mentioned	2	1
2	High intensity cool white light used	Fluorescent F72T12/CW/HO	Fluorescent daylight Phillips TLD 18 W/54
3	Initial concentration of PMS used	0.5 mg/ml in 0.01 M TRIS–HCl	1 mg/ml in 0.01 M TRIS–HCl
4	Starting pH	7	7.4
5	Photolysis time	4 days	2.5 h
6	Rotary evaporation	Yes	No
7	Lyophilization of photolysed products	Yes	No
8	Sequential acidification/alkalinization, and chloroform extraction-purification	No	Yes (3 times)
9	Hexane precipitation from chloroform, filtration, methanol elution from filter	No	Yes
10	TLC plate	Silica gel 60 column (2.5 cm × 82 cm)	Merck – HPTLC Pre-coated Silica Gel 60 Plates
11	Chloroform:methanol proportion for TLC plate equilibration	99:1	0:100
12	Solvent for pyocyanin loading on TLC plate	Methanol	Chloroform
13	Chloroform:methanol proportion for pyocyanin elution	85:15	0:100
14	Lyophilization after pyocyanin elution	Yes	No
15	Pyocyanin yield from TLC plate	60%	>90%
16	Purification steps	+	++++
17	Total time for extraction and purification	++++	+

The key procedural differences between the original protocol and our exhaustively revised protocol are tabulated here. Our procedure substantially decreases the total time taken for the synthesis, extraction and purification of pyocyanin, and increases the pyocyanin proportion produced in the final step. Our procedure involves multiple extraction/re-extraction steps, and more purification steps. It removes a rotary evaporation step and multiple lyophilization steps.
